# Omics on bioleaching: current and future impacts

**DOI:** 10.1007/s00253-015-6903-8

**Published:** 2015-08-18

**Authors:** Patricio Martinez, Mario Vera, Roberto A. Bobadilla-Fazzini

**Affiliations:** BioSigma ‘S.A.’, Parque Industrial Los Libertadores, Lote 106, Colina, Chile; Biofilm Centre, Aquatische Biotechnologie, Universität Duisburg-Essen, Universitätstraße 5, 45141 Essen, Germany

**Keywords:** Bioleaching, Genomics, Proteomics, Metabolomics

## Abstract

Bioleaching corresponds to the microbial-catalyzed process of conversion of insoluble metals into soluble forms. As an applied biotechnology globally used, it represents an extremely interesting field of research where *omics* techniques can be applied in terms of knowledge development, but moreover in terms of process design, control, and optimization. In this mini-review, the current state of genomics, proteomics, and metabolomics of bioleaching and the major impacts of these analytical methods at industrial scale are highlighted. In summary, genomics has been essential in the determination of the biodiversity of leaching processes and for development of conceptual and functional metabolic models. Proteomic impacts are mostly related to microbe-mineral interaction analysis, including copper resistance and biofilm formation. Early steps of metabolomics in the field of bioleaching have shown a significant potential for the use of metabolites as industrial biomarkers. Development directions are given in order to enhance the future impacts of the omics in biohydrometallurgy.

## Introduction

The simultaneous study of the complete set of genetic, protein, and metabolic material of living things will probably lead science to decipher at least some of the mysteries of life. As genomics is not the only responsible for life’s complexity, more “-omes” are required to solve this puzzle (Ball 2013), and therefore, we must account for the “dark matter” of the biological universe (Varki 2013). Bioleaching, known as the microbial-catalyzed process of conversion of insoluble metal sulfides into soluble forms, has introduced the classical genomics for a while, as well as the dark matter omics, such as proteomics and metabolomics more recently, with great advances in knowledge and several more to be accomplished.

During the 1940s, the phenomenon of metal sulfide dissolution in an acidic medium was first attributed to the action of certain microorganisms (Colmer and Hinkle 1947). This was the first step in applying microbiology to mining and opened the door for research in acidophilic microorganisms among this field. Decades after this breakthrough, recovery of metals from sulfide ores employs several different types of extremophiles, including bacterial and archaeal species. In addition, extremophiles are also being studied to understand life in extreme conditions, and therefore, the study of proteomics and metabolomics will render the discovery of biomarkers used in the search for evidence of existing or past extra-terrestrial life (Bonnefoy and Holmes 2012). Extremophilic microorganisms have been isolated from mining operations (Johnson et al. 2001; Okibe et al. 2003; Tyson et al. 2004), and their role in the dynamics and evolution of minerals has been widely discussed (Santelli et al. 2009). These microorganisms, naturally present in the native flora of minerals, are known to have a role in the biohydrometallurgical leaching processes, and their presence has been linked to improved extraction of metals including copper, nickel, cobalt, zinc, and uranium. Bioleaching dumps and heaps operating with this technology can be found in mines all over the world such as Radomiro Tomic (Chile), Girilambone (Australia), Barberton (South Africa), Cerro Verde (Peru), Morenci (United States), Talvivaara (Finland), Sabetaung-Kyisintaung (Myanmar), and Dexing and Zijinshan (China), among other mining sites. Therefore, *omics* techniques have a unique worldwide-applied biotechnological niche to be impacted not only in terms of knowledge development but also for biohydrometallurgical process design, control, and optimization.

In this mini-review, differing from others previously published (Cárdenas et al. 2010), we summarize the state of genomics, proteomics, and metabolomics in the field of acidophiles, emphasizing the current and future impacts of this biotechnological knowledge in the development of bioleaching at industrial scale.

## Current and future impacts of genomics on bioleaching

**Fig. 1:**
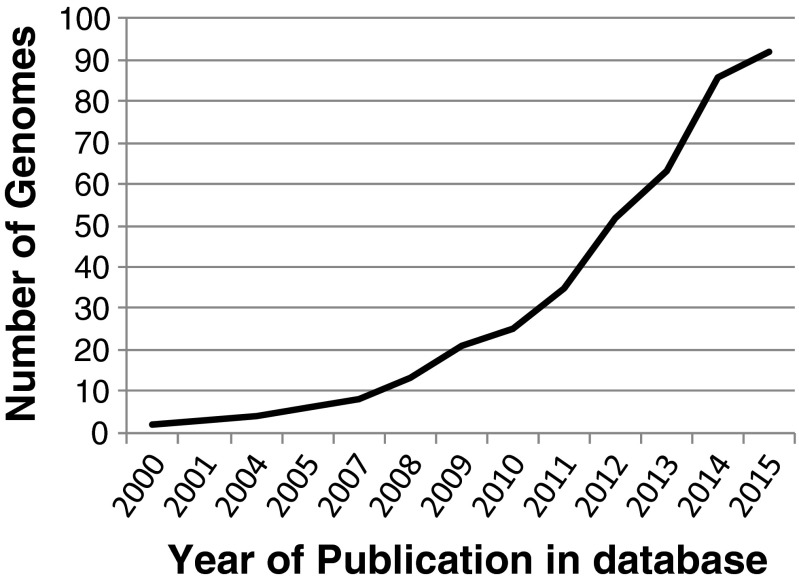
Increment in the number of publicly available complete genomes of microorganisms involved in bioleaching according to the NCBI database from year 2000–2015 (http://www.ncbi.nlm.nih.gov/genome/browse/)

Today, we have a completely different scenario compared with the year 2000, when the first gapped genome sequence of *Acidithiobacillus ferrooxidans* (formerly *Thiobacillus ferrooxidans* and the archetype of a bioleaching microorganism) was published (Selkov et al. 2000). Currently, 55 bacterial and 36 archaeal complete genomes from microorganisms that are present in bioleaching processes are publicly available according to the NCBI database (see Table 1), and the tendency to an exponential increase is evident (see Fig. 1). One main question is how the knowledge generated in these 15 years of genomics of leaching microorganisms has contributed to improvements in this field and particularly, which are and would be the major future impacts at industrial scale.

**Table 1:** List of publicly available genomes of microorganisms involved in bioleaching according to the NCBI database

Organism/name	Kingdom	Group	Subgroup	Size (Mb)	Chr	Plasmids	GC (%)	Reference
*Thermoplasma acidophilum* DSM 1728	*Archaea*	*Euryarchaeota*	*Thermoplasmata*	1.56491	1	1	46.0	Urbieta et al. (2014)
*Thermoplasma volcanium* GSS1	*Archaea*	*Euryarchaeota*	*Thermoplasmata*	1.5848	1	–	39.9	You et al. (2011)
*Sulfolobus solfataricus* P2	*Archaea*	*Crenarchaeota*	*Thermoprotei*	2.99225	1	3	35.8	Mardanov et al. (2010)
*Picrophilus torridus*	*Archaea*	*Euryarchaeota*	*Thermoplasmata*	1.5459	1	–	36.0	Lucas et al. (2012)
*Sulfolobus acidocaldarius* DSM 639	*Archaea*	*Crenarchaeota*	*Thermoprotei*	2.22596	1	–	36.7	Liu et al. (2011)
*Sulfolobus tokodaii*	*Archaea*	*Crenarchaeota*	*Thermoprotei*	2.69476	1	–	32.8	Auernik et al. (2008)
*Ferroplasma acidarmanus*	*Archaea*	*Euryarchaeota*	*Thermoplasmata*	1.93521	1	–	36.5	Lucas et al. (2012)
*Metallosphaera sedula*	*Archaea*	*Crenarchaeota*	*Thermoprotei*	2.19152	1	–	46.2	Chen et al. (2005)
*Sulfolobus islandicus* L.S.2.15	*Archaea*	*Crenarchaeota*	*Thermoprotei*	2.73627	1	12	35.1	Mao et al. (2012)
*Sulfolobus islandicus* M.14.25	*Archaea*	*Crenarchaeota*	*Thermoprotei*	2.60883	1	12	35.1	Mao et al. (2012)
*Sulfolobus islandicus* M.16.27	*Archaea*	*Crenarchaeota*	*Thermoprotei*	2.6924	1	12	35.0	Mao et al. (2012)
*Sulfolobus islandicus* M.16.4	*Archaea*	*Crenarchaeota*	*Thermoprotei*	2.58665	1	12	35.0	Guo et al. (2011)
*Sulfolobus islandicus* Y.G.57.14	*Archaea*	*Crenarchaeota*	*Thermoprotei*	2.70206	1	12	35.4	Reno et al. (2009)
*Sulfolobus islandicus* Y.N.15.51	*Archaea*	*Crenarchaeota*	*Thermoprotei*	2.85441	1	12	35.3	Jaubert et al. (2013)
*Acidilobus saccharovorans*	*Archaea*	*Crenarchaeota*	*Thermoprotei*	1.49645	1	–	54.2	Reno et al. (2009)
*Acidianus hospitalis*	*Archaea*	*Crenarchaeota*	*Thermoprotei*	2.13765	1	1	34.1	Cadillo-Quiroz et al. (2012)
*Metallosphaera cuprina*	*Archaea*	*Crenarchaeota*	*Thermoprotei*	1.84035	1	–	42.0	Cadillo-Quiroz et al. (2012)
*Sulfolobus islandicus* REY15A	*Archaea*	*Crenarchaeota*	*Thermoprotei*	2.52299	1	12	35.3	Reno et al. (2009)
*Sulfolobus islandicus* HVE10/4	*Archaea*	*Crenarchaeota*	*Thermoprotei*	2.6552	1	12	35.1	Reno et al. (2009)
*Caldisphaera lagunensis*	*Archaea*	*Crenarchaeota*	*Thermoprotei*	1.54685	1	–	30.0	Cadillo-Quiroz et al. (2012)
*Metallosphaera yellowstonensis*	*Archaea*	*Crenarchaeota*	*Thermoprotei*	2.81745	–	–	47.7	Cadillo-Quiroz et al. (2012)
*Sulfolobus acidocaldarius* N8	*Archaea*	*Crenarchaeota*	*Thermoprotei*	2.22596	1	–	36.7	Cadillo-Quiroz et al. (2012)
*S. acidocaldarius* Ron12/I	*Archaea*	*Crenarchaeota*	*Thermoprotei*	2.22596	1	–	36.7	Guo et al. (2011)
*Sulfolobus islandicus* M.16.2	*Archaea*	*Crenarchaeota*	*Thermoprotei*	2.64525	1	12	35.0	Reno et al. (2009)
*S. islandicus* M.16.23	*Archaea*	*Crenarchaeota*	*Thermoprotei*	2.60113	1	12	35.1	Reno et al. (2009)
*S. islandicus* M.16.40	*Archaea*	*Crenarchaeota*	*Thermoprotei*	2.66478	1	12	35.0	Lucas et al. (2012)
*S. islandicus* M.16.43	*Archaea*	*Crenarchaeota*	*Thermoprotei*	2.58984	1	12	35.0	McCarthy et al. (2015)
*S. islandicus* M.16.47	*Archaea*	*Crenarchaeota*	*Thermoprotei*	2.63756	1	12	35.1	She et al. (2001)
*S. solfataricus* 98/2	*Archaea*	*Crenarchaeota*	*Thermoprotei*	2.66897	1	3	35.8	McCarthy et al. (2015)
*S. acidocaldarius* SUSAZ	*Archaea*	*Crenarchaeota*	*Thermoprotei*	2.22596	1	–	36.3	McCarthy et al. (2015)
*S. islandicus* LAL14/1	*Archaea*	*Crenarchaeota*	*Thermoprotei*	2.46518	1	12	35.1	Kawarabayasi et al. (2001)
*Acidianus copahuensis*	*Archaea*	*Crenarchaeota*	*Thermoprotei*	2.45402	–	–	35.6	Bulaev (2015)
*S. solfataricus* SULB	*Archaea*	*Crenarchaeota*	*Thermoprotei*	2.72726	1	3	35.9	Allen et al. (2007)
*S. solfataricus* 98/2 SULC	*Archaea*	*Crenarchaeota*	*Thermoprotei*	2.72731	1	3	35.9	Fütterer et al. (2004)
*S. solfataricus* SULA	*Archaea*	*Crenarchaeota*	*Thermoprotei*	2.72734	1	3	35.9	Ruepp et al. (2000)
*Acidiplasma* sp. MBA-1	*Archaea*	*Euryarchaeota*	*Thermoplasmata*	1.74736	–	–	34.0	Kawashima et al. (2000)
*Acidiphilium cryptum* JF-5	*Bacteria*	*Proteobacteria*	*Alphaproteobacteria*	3.96308	1	8	67.1	Clum et al. (2009)
*Alicyclobacillus acidocaldarius* LAA1	*Bacteria*	*Firmicutes*	*Bacilli*	2.94328	–	–	61.7	Poehlein et al. (2015)
*Anoxybacillus flavithermus* WK1	*Bacteria*	*Firmicutes*	*Bacilli*	2.84675	1	–	41.8	Kyrpides et al. (2014)
*Acidithiobacillus ferrooxidans* ATCC 23270	*Bacteria*	*Proteobacteria*	*Gammaproteobacteria*	2.9824	1	2	58.8	Lucas et al. (2008)
*Acidithiobacillus ferrooxidans* ATCC 53993	*Bacteria*	*Proteobacteria*	*Gammaproteobacteria*	2.88504	–	–	58.9	Mavromatis et al. (2010)
*Acidimicrobium ferrooxidans* DSM 10331	*Bacteria*	*Actinobacteria*	*Actinobacteria*	2.15816	1	–	68.3	Chen et al. (2011)
*Acidithiobacillus caldus* ATCC 51756	*Bacteria*	*Proteobacteria*	*Gammaproteobacteria*	2.98705	1	4	61.4	Shemesh et al. (2013)
*A. acidocaldarius* subsp. *acidocaldarius* DSM 446	*Bacteria*	*Firmicutes*	*Bacilli*	3.20569	1	3	61.9	Kyrpides et al. (2013)
*Acidiphilium multivorum* AIU301	*Bacteria*	*Proteobacteria*	*Alphaproteobacteria*	4.21474	–	–	67.0	Kyrpides et al. (2013)
*Acidithiobacillus* sp. GGI-221	*Bacteria*	*Proteobacteria*	*Gammaproteobacteria*	3.17493	–	–	58.6	Wang et al. (2012)
*A. acidocaldarius* subsp. *acidocaldarius* Tc-4-1	*Bacteria*	*Firmicutes*	*Bacilli*	3.12405	–	–	61.5	King et al. (2014)
*Sulfobacillus acidophilus* TPY	*Bacteria*	*Firmicutes*	*Clostridia*	3.55121	1	–	56.8	Kyrpides et al. (2013)
*Leptospirillum ferriphilum* ML-04	*Bacteria*	*Nitrospirae*	*Nitrospira*	2.40616	1	–	54.6	Belduz et al. (2015)
*Acidithiobacillus caldus* SM-1	*Bacteria*	*Proteobacteria*	*Gammaproteobacteria*	3.2376	1	4	60.9	Rozanov et al. (2014)
*Acidithiobacillus ferrivorans* SS3	*Bacteria*	*Proteobacteria*	*Gammaproteobacteria*	3.20755	–	–	56.6	Rajan et al. (2013)
*Acidithiobacillus thiooxidans* ATCC 19377	*Bacteria*	*Proteobacteria*	*Gammaproteobacteria*	3.01987	–	–	53.2	Matsutani et al. (2013)
*Alicyclobacillus hesperidum* URH17-3-68	*Bacteria*	*Firmicutes*	*Bacilli*	2.96716	–	–	53.8	Wang et al. (2014)
*Anoxybacillus* sp. DT3-1	*Bacteria*	*Firmicutes*	*Bacilli*	2.59664	–	–	41.4	Caspers et al. (2013)
*Anoxybacillus* sp. SK3-4	*Bacteria*	*Firmicutes*	*Bacilli*	2.67159	–	–	41.9	Saw et al. (2008)
*Anoxybacillus kamchatkensis* G10	*Bacteria*	*Firmicutes*	*Bacilli*	2.85866	–	–	41.4	Belduz et al. (2014)
*Sulfobacillus acidophilus* DSM 10332	*Bacteria*	*Firmicutes*	*Clostridia*	3.55783	1	–	56.8	Lee et al. (2012)
*Sulfobacillus thermosulfidooxidans* str. *Cutipay*	*Bacteria*	*Firmicutes*	*Clostridia*	3.86201	1	–	49.3	Filippidou et al. (2014)
*Leptospirillum ferrooxidans* C2-3	*Bacteria*	*Nitrospirae*	*Nitrospira*	2.55954	–	–	50.0	Patel (2015)
*Alicyclobacillus acidoterrestris* ATCC 49025	*Bacteria*	*Firmicutes*	*Bacilli*	4.06355	–	–	52.2	Goh et al. (2014)
*Alicyclobacillus contaminans* DSM 17975	*Bacteria*	*Firmicutes*	*Bacilli*	3.27232	–	–	58.2	Bryanskaya et al. (2014)
*Alicyclobacillus herbarius* DSM 13609	*Bacteria*	*Firmicutes*	*Bacilli*	3.31051	–	–	57.2	Kahar et al. (2013)
*Alicyclobacillus pomorum* DSM 14955	*Bacteria*	*Firmicutes*	*Bacilli*	3.39856	–	–	53.7	King et al. (2014)
*A. flavithermus* TNO-09.006	*Bacteria*	*Firmicutes*	*Bacilli*	2.65842	–	–	41.8	Poli et al. (2015)
*A. flavithermus* AK1	*Bacteria*	*Firmicutes*	*Bacilli*	2.63066	–	–	42.7	Anderson et al. (2012)
*A. flavithermus* NBRC 109594	*Bacteria*	*Firmicutes*	*Bacilli*	2.77262	–	–	41.7	Li et al. (2011)
*Sulfobacillus thermosulfidooxidans* ST	*Bacteria*	*Firmicutes*	*Clostridia*	3.33355	1	–	48.3	Guo et al. (2013)
*Leptospirillum* sp. group IV “UBA BS”	*Bacteria*	*Nitrospirae*	*Nitrospira*	1.52216	–	–	59.0	Travisany et al. (2012)
*Ferrimicrobium acidiphilum* DSM 19497	*Bacteria*	*Actinobacteria*	*Actinobacteria*	3.08865	–	–	55.3	Cárdenas et al. (2014)
*Alicyclobacillus macrosporangiidus* CPP55	*Bacteria*	*Firmicutes*	*Bacilli*	4.09165	–	–	62.3	Mi et al. (2011)
*Anoxybacillus* sp. KU2-6(11)	*Bacteria*	*Firmicutes*	*Bacilli*	2.8835	–	–	41.7	Guo et al. (2014)
*Anoxybacillus* sp. BCO1	*Bacteria*	*Firmicutes*	*Bacilli*	2.8087	–	–	41.7	Fujimura et al. (2012)
*Anoxybacillus* sp. ATCC BAA-2555	*Bacteria*	*Firmicutes*	*Bacilli*	6.97598	–	–	46.8	Goltsman et al. (2013)
*Anoxybacillus ayderensis*	*Bacteria*	*Firmicutes*	*Bacilli*	2.83235	–	–	41.8	Liu and Yang (2014)
*A. flavithermus* subsp. *yunnanensis* str. E13	*Bacteria*	*Firmicutes*	*Bacilli*	2.83839	–	–	41.4	Kyrpides et al. (2014)
*A. flavithermus*	*Bacteria*	*Firmicutes*	*Bacilli*	2.83868	–	–	42.3	Copeland et al. (2007)
*Anoxybacillus gonensis*	*Bacteria*	*Firmicutes*	*Bacilli*	2.75833	–	–	41.4	Hosoyama et al. (2010)
*Anoxybacillus tepidamans* PS2	*Bacteria*	*Firmicutes*	*Bacilli*	3.364	–	–	43.0	Poehlein et al. 2014
*Anoxybacillus thermarum*	*Bacteria*	*Firmicutes*	*Bacilli*	2.73691	–	–	42.0	San Martin-Uriz et al. (2011)
*Leptospirillum ferriphilum* YSK	*Bacteria*	*Nitrospirae*	*Nitrospira*	2.33059	–	–	54.5	Moya-Beltrán et al. (2014)
*L. ferriphilum*	*Bacteria*	*Nitrospirae*	*Nitrospira*	2.40588	–	–	54.1	Valdes et al. (2009)
*Acidicaldus organivorans*	*Bacteria*	*Proteobacteria*	*Alphaproteobacteria*	2.98963	–	–	68.5	You et al. (2011)
*Acidiphilium* sp. PM	*Bacteria*	*Proteobacteria*	*Alphaproteobacteria*	3.92946	–	–	66.4	Talla et al. (2014)
*Acidiphilium* sp. JA12-A1	*Bacteria*	*Proteobacteria*	*Alphaproteobacteria*	4.18836	–	–	66.9	Liljeqvist et al. (2011)
*Acidiphilium angustum* ATCC 35903	*Bacteria*	*Proteobacteria*	*Alphaproteobacteria*	4.17541	1	8	63.6	Valdés et al. (2008)
*Ferrovum myxofaciens*	*Bacteria*	*Proteobacteria*	*Betaproteobacteria*	2.70219	–	–	54.3	Lucas et al. (2008)
*Acidithiobacillus ferrivorans*	*Bacteria*	*Proteobacteria*	*Gammaproteobacteria*	3.43367	–	–	56.4	Bagatharia et al. (2010)
*Acidithiobacillus ferrooxidans*	*Bacteria*	*Proteobacteria*	*Gammaproteobacteria*	4.18422	–	–	57.6	Travisany et al. (2014)
*Acidithiobacillus thiooxidans* A01	*Bacteria*	*Proteobacteria*	*Gammaproteobacteria*	3.82016	–	–	53.1	Yin et al. (2014)
*A. thiooxidans*	*Bacteria*	*Proteobacteria*	*Gammaproteobacteria*	3.9379	–	–	52.8	Valdes et al. (2011)
*Thiobacillus prosperus* DSM 5130	*Bacteria*	*Proteobacteria*	*Gammaproteobacteria*	3.32321	–	–	64.5	Ossandon et al. (2014)
*Acidithrix ferrooxidans*	*Bacteria*	*Actinobacteria*	*Actinobacteria*	4.01987	–	–	47.7	Reference

Genomics has certainly made a major impact on our knowledge of bioleaching. First of all, partial and full genome sequencing has allowed the determination of the biodiversity within leaching environments and the development of molecular-based methods to scrutinize the temporal population dynamics of different bioleaching processes. For a long time, *Acidithiobacillus ferrooxidans* was thought to be the most significant microorganism for metal sulfide bioleaching, however, the advances in genomics knowledge together with the development of bioidentification molecular techniques such as DGGE, FISH, and quantitative PCR (qPCR) has driven the exploration of extreme mineral leaching environments for new microorganisms with potential commercial applications, resulting in a more comprehensive understanding of the biodiversity of acidophilic environments. Interesting examples are the biodiversity obtained from heap solids and solutions collected from the Myanmar Ivanhoe Copper company, which showed the prevalence of heterotrophic Archaea initially classified as *Ferroplasma cupricumulans* (Hawkes et al. 2006) and later re-classified as *Acidiplasma cupricumulans* (Golyshina et al. 2009). Also, the identification of moderate thermophilic mixo- and/or heterotrophs from the *Sulfobacillus* genus as the dominant population of the heap operation at the Agnes Gold Mine in Barberton, South Africa (Coram-Uliana et al. 2006), or the presence of chemolithoautotrophic iron-oxidizing Leptospirilli in a tailings impoundment at the La Andina copper mine in Chile (Diaby et al. 2007). These studies, among others, have shown the diversity of microbial populations in bioleaching operations. In our research group, we have used genomics information as the base for the development of patented techniques that allow an accurate monitoring of the microbial populations during the bioleaching process (Maass et al. 2010; Parada et al. 2013; Ehrenfeld et al. 2012). Moreover, spatial and temporal microbial population dynamics changes have been described within and during bioelaching stages (Remonsellez et al. 2009; Acosta et al. 2014) and also depending on the oxidation state of the minerals (Schippers et al. 2010). In our opinion, the spatio-temporal analysis of acidophilic microbial populations during bioleaching is a major impact that (meta)-genomics will generate in the coming future. Recently, we have analyzed the microbial dynamics of lab-scale bioleaching of a mainly primary copper sulfide low-grade ore by massive sequencing technology using the MiSeq Illumina platform. Besides the prevalence of *Acidithiobacillus* and *Sulfobacillus* genera, the presence of anoxybacilli as relevant members of the leaching microbial community has been identified. *Anoxybacillus* spp., initially named as strict anaerobes, includes aerobes, facultative anaerobes, and facultative aerobes found in moderate- to high-temperature habitats such as geothermal hot springs (Goh et al. 2014). Despite that this genus has been characterized so far as alkaliphilic or alkali tolerant, our massive sequencing data has recently shown that up to 13 % of the acid-leaching population is composed of this genus under the conditions tested (Bobadilla-Fazzini, unpublished data).

Future impacts of genomics, by means of more comprehensive, reliable, fast, and cheap next-generation sequencing technologies will lead to a better bioleaching operational design and control (Demergasso et al. 2010).

A second major impact of genomics has been to provide information for the in silico reconstruction of metabolic capabilities of formerly unknown essential aspects of bioleaching, such as the metabolic routes for iron and reduced inorganic sulfur compounds (RISC) oxidation. Conceptual models based on genomics data have shown the intricate and alternative metabolic pathways for RISC compounds showing common features between Bacteria and Archaea (Chen et al. 2012), new metabolic characteristics, and environmental adaptation in *Acidithiobacillus ferrooxidans* and *Leptospirillum ferriphilum* (Levicán et al. 2008), *Acidithiobacillus caldus* (Mangold et al. 2011), and *Sulfobacillus thermosulfidooxidans* (Guo et al. 2014; Justice et al. 2014). Genomics comparisons have shown major differences between different strains of the same species. *Ferroplasma acidarmanus*-related population exhibits a mosaic genome structure in which a small number of sequence types predominate, suggesting that such structure may be a common feature of natural archaeal populations (Allen et al. 2007). *Acidithiobacillus ferrooxidans* ATCC 23270^T^ and ATCC 53993 have 2397 genes in common, which represent between 78 and 90 % of their genomes, being particularly different in terms of a genomic island which provides higher copper resistance to strain ATCC 53993 (Orellana and Jerez 2011). Further genetic multilocus sequence analysis (MLSA) of 21 strains has shown that iron-oxidizing acidithiobacilli are subdivided into at least four distinct taxa (Amouric et al. 2011). *Acidithiobacillus thiooxidans* strains ATC19377, Licanantay, and A01 share only 75 to 89 % of their genomes among them (Travisany et al. 2014), and *Sulfolobus* genomes within the same species vary between 18 and 28 % (Guo et al. 2011). In this context, these genomic differences are not far from those observed between other acidophilic species, e.g., *Picrophilus torridus* shares 66 and 58 % of its genome with *Thermoplasma acidophilum* and *Sulfolobus solfataricus* genomes, respectively (Fütterer et al. 2004). All these data points out that new species will probably arise concomitantly with further refinements of genomics data generation and analysis.

In addition, more holistic conceptual metabolic models have been built based on genomics data for *Acidilobus saccharovorans* (Mardanov et al. 2010), *Metallosphaera sedula* (Auernik et al. 2008), and *Acidianus hospitalis* (You et al. 2011). Even further, more sophisticated genome-scale models are available for *Acidithiobacillus ferrooxidans* (Hold et al. 2009), *Acidithiobacillus thiooxidans* (Bobadilla-Fazzini et al. 2013), and *Leptospirillum ferrooxidans* (Merino et al. 2010). These genome-scale metabolic models are not only conceptual but also functional, allowing accurate predictions of metabolic fluxes and expected biomass yields, being novel tools to be used in biohydrometallurgical as well as environmental applications. Recently, a metabolic reconstruction of a simplified bioleaching consortium formed by *L. ferriphilum* and *Ferroplasma acidiphilum* is available (Merino et al. 2015), and despite that it requires further development, it can predict which species could prevail under a specific culture condition. Future impacts point towards metabolic functional models able to predict the behavior of microbial leaching communities in order to design new strategies to enhance and optimize productivity in bioleaching processes.

## Current and future impacts of proteomics on bioleaching

As with genomics, proteomics has impacted the understanding of the microbe-mineral interaction. Perhaps not dark but “gray biological matter,” the protein complement or proteome of acidophilic microbial cells constitute a key element within the omics analytical methods. Despite the physicochemical nature of most leaching mechanisms described so far (Rawlings 2002), several studies have demonstrated that protein-related carriers present in the extracellular polymeric substance (EPS) layers are able to accumulate colloidal sulfur and enhance the bioleaching of metal sulfides (Tributsch 2001; Bobadilla-Fazzini et al. 2011). Essential parts of the microbial role in bioleaching processes are the iron and RISC oxidation, which occur mainly in the extracellular space or in the periplasmic/cytoplasmic space, respectively. In order to achieve a better understanding of the sulfur oxidation metabolism in *Acidithiobacillus ferrooxidans* ATCC 23270^T^, a high-throughput proteOMIC study revealed the presence of 131 proteins in the periplasmic fraction of thiosulfate-grown cells. Among these, 86 % of the protein possessed predicted export/secretion signals. Functional categories such as transport and binding proteins, cell envelope, energy metabolism, as well as cell and protein folding accounted for 45 % of the total identified proteins. Hypothetical proteins and unique unknown proteins accounted for 36 % of the identified proteins, and their functions remain until now to be elucidated (Chi et al. 2007). Future impacts of proteomics must be directed towards the functional characterization of these new proteins, a difficult task that will probably render potential new biotechnological additives for industrial applications.

Another impact of proteomics in the understanding of bioleaching is related to heavy metal resistance. *Acidithiobacillus ferrooxidans* ATCC 23270^T^ is known to tolerate high concentrations of copper (>100 mM copper sulfate). Two high-throughput proteomics studies have described the global proteOMIC responses of this bacterium against copper stress. The molecular adaptations of *Acidithiobacillus ferrooxidans* to the presence of copper included an upregulation of RND-type Cus systems, an increase in proteins related to cysteine and hystidine biosynthesis as well as putative disulfide isomerases, probably involved in repair mechanisms for damaged disulfide bonds upon copper exposure. Downregulation of the major outer membrane protein (Omp40) and some ionic transporters suggests that a decrease in the influx of metal and other cations might occur upon copper exposure (Almárcegui et al. 2014a, b). Interestingly, the proteins encoded in the *rus* operon, involved in the transfer of electrons obtained from iron oxidation, were found to be increased in copper-grown cells, suggesting their participation in a copper-resistance mechanism, and supporting the role of rusticyanin (Rus) as a copper chaperone (Almárcegui et al. 2014b).

Biofilm formation is a central aspect of bioleaching. Biofilm cells are embedded in a matrix of EPS which plays an important role in attachment to solid surfaces and their further corrosion (Gehrke et al. 1998; Vera et al. 2013a). To this respect, the first high-throughput proteomic study of the biofilm formation in *Acidithiobacillus ferrooxidans* ATCC 23270^T^ was done in order to map the changes during its early biofilm formation process. Proteomes from planktonic and sessile cells upon 24 h of biofilm formation on pyrite were compared using semi-quantitative shotgun proteomics. Several molecular adaptations for growing on pyrite surfaces were observed to occur in the biofilm subpopulation. Among these, membrane and outer membrane transport functions, including increased levels of proteins involved in EPS biosynthesis, efflux pumps, lipoproteins, ABC transporters, and proteins related to stress response were predominant (Vera et al. 2013b). Changes in proteins related to responses against osmotic and oxidative stress were also detected. Apart from the osmolarity sensor protein EnvZ (one of the most induced proteins found in this study), a protein encoding an iron and 2-oxoglutarate (2-OG)-dependent dioxygenase (AFE_3138) was also found to have increased levels in biofilm cells. As a response to high osmolarity, many microorganisms synthesize various types of compatible solutes, such as ectoine. Paralogs of the 2-OG-dependent dioxygenase family have been shown to be involved in their biosynthesis (Reuter et al. 2010). In addition, several proteins involved in glutathione (GSH) metabolism were found to be induced in biofilm cells. GSH is involved in responses against oxidative stress and RISC oxidation, catalyzed by a periplasmic sulfur dioxygenase (SDO) (Rohwerder and Sand 2003). A subunit of an ATP-dependent GSH transporter (CydC), a GSH reductase (GR), and a protein of the YghU family of GSH S-transferases were enhanced in biofilms. The data suggest an increase in periplasmic GSH levels. These are probably related to the transport of sulfur moieties for RISC oxidation and/or also to a GSH-driven response against oxidative stress, which occurs in biofilm cells upon contact to pyrite (Vera et al. 2013b). In this context, it has been shown that the GR encoding gene is induced after *Acidithiobacillus ferrooxidans* is exposed to copper, suggesting its involvement in recovering GSH pools and cell homeostasis (Xia et al. 2011). Also an enhanced biosynthesis of cofactors and coenzymes was suggested to occur in *Acidithiobacillus ferrooxidans* biofilms. These include proteins containing iron-sulfur clusters and others involved in iron acquisition, heme, pyrroloquinolinequinone (PQQ) and ubiquinone metabolism. Of 1319 proteins detected in this study, 231 were hypothetical, and 12 % of them were found to be differentially regulated in biofilm cells (Vera et al. 2013b). More exhaustive proteomic studies of biofilm formation on different surfaces, including extracellular or EPS proteomes, will greatly impact in a better biohydrometallurgical performance.

In the frame of the discovery and use of biomarkers, proteomics data have been used to infer the genomic type of natural populations in an acid mine drainage (AMD) community sample. Samples from two locations (UBA and five-way GC) within the Richmond Mine, Iron Mountain, California, were each dominated by a distinct *Leptospirillum* group II type, differing by 0.3 % at the 16S rRNA sequence level. Both genomes share 80 % of their genes with 95.2 % average amino acid identity. A proteomics-inferred genome typing (PIGT), provided evidence of recombination among these two closely related *Leptospirillum* group II populations (Lo et al. 2007). Later on, PIGT has been used to genotype the dominant *Leptospirillum* group II population in 27 biofilm samples from the Richmond Mine over a 4-year period. Six distinct phenotypes, which are recombinants derived from two parental genotypes, were identified confirming that homologous recombination is used as a strategy for fine scale environmental adaptation within those biofilms (Denef et al. 2009). Proteomics analyses of these biofilms have been also performed with laboratory-cultivated biofilms, inoculated with these environmental samples (Belnap et al. 2010, 2011). Interestingly, laboratory-grown biofilms were also dominated by *Leptospirillum* group II, with a lower abundance of *Leptospirillum* group III. A proteomic comparison was done between field and laboratory biofilms. Proteins related to functional categories such as energy production and conversion, cell motility, cell wall/membrane/envelope biogenesis, and intracellular trafficking, as well as secretion and vesicular transport were found to be more abundant in field biofilm samples while transcriptional proteins as well as proteins probably involved in defense mechanisms were more abundant in laboratory-grown biofilms (Belnap et al. 2010). Further studies determined that *Leptospirillum* group II proteins involved in amino acid and nucleotide metabolism, as well as cell membrane/envelope biogenesis were overrepresented at high pH. In addition, a pH-specific niche partitioning was shown to occur for some low-abundance bacteria and archaea, since *Leptospirillum* group III was more abundant in biofilms grown at higher pH values, whereas archaeal species were more abundant at lower pH values (Belnap et al. 2011). In this context, the use of proteomics as a tool to analyze the microbial biodiversity and their specific adaptations within field operations will strongly impact the monitoring and control of industrial bioleaching processes in the near future.

By high-throughput proteomics and metabolomics it has been shown that the identified proteins and metabolites from *Leptospirillum* groups II and III exhibit organism-related correlation patterns, which suggest a restructuring of their metabolic and/or regulatory networks which would reduce their competition and allow them to occupy distinct niches (Wilmes et al. 2010). After evaluation of the reproducibility of AMD community proteomes, a set of reliable classifier proteins was identified which may be used to predict growth stages of biofilm communities. During early stages of biofilm growth, *Leptospirillum* group II cell population responded to some abiotic stresses by reorganizing their metabolism, since enzymes involved in protein biosynthesis, cell division, and utilization of one or two carbon compounds were found to be more abundant. Although the abundances of proteins did not change much between early and intermediate samples, significant changes occurred as the biofilm matures in later growth stages. Stress responses were more abundant in early stage biofilms, since proteins involved in metal efflux were found to be increased. Among the increased proteins in this growth stage, a cytochrome 572, a cytochrome oxidase with high molecular oxygen affinity, as well as a cytochrome c-553 were found. Cell division functions were more abundant in early stage biofilms and these seem to decrease because of an accumulation of DNA mutations. This was in agreement with a 26-fold increase in the abundance of ribosomal proteins detected in early stage biofilms, if compared with the late stage. Several enzymes involved in the metabolism of more complex carbohydrates, amino acid biosynthesis, amino- and nucleotide-sugar metabolism, lipopolysaccharide biosynthesis, starch, and threhalose metabolism were more abundant in mature biofilms. Proteomes from late-stage biofilms also showed an increased level of proteins involved in phosphate and molybdenum transport, suggesting that essential nutrients such as oxygen, phosphorous, nitrogen, and molybdenum become limiting in those biofilms (Mueller et al. 2011). The response of these AMD microbial biofilm communities to temperature gradients has recently been studied. Cultivation at elevated temperatures, e.g., increase from 40 to 46 °C repressed carbon fixation in two *Leptospirillum* genotypes while a third one was probably subjected to a viral stress, which would increase the carbon turnover through the release of the viral lysate (Mosier et al. 2015). Several enzymes upregulated could probably be involved in changes in the EPS composition in these AMD biofilms, which have been shown to possess carbohydrates such as glucose, galactose, rhamnose, heptose and mannose (Jiao et al. 2010). Recently, it has been shown that approximately 29 % of the proteins reliably detected in the dominant *Leptospirillum* type II biofilm population carry post-translational modifications (PTMs), and among these, 43 % carry more than one PTM. The PTMs profile strongly differs between early and mature biofilms, as well as between orthologous of two ecologically differentiated *Leptospirillum* group II bacteria (Li et al. 2014).

Future proteomics studies focused on new species isolated from biomining operations, their biofilm formation (considering spatial and temporal population dynamics), as well as interspecies interactions, will provide new knowledge for the optimization of bioleaching at industrial scale.

## Current and future impacts of metabolomics on bioleaching

Moving towards the “biological dark matter,” metabolomics has allowed the global study of disturbances in the metabolism of microorganisms (Ishii et al. 2007), which are directly related to phenotype and environmental conditions. The oxidizing capability of biomining microbes is the driving force of bioleaching, and thus research efforts have focused on sulfur and iron oxidation. A network of cellular metabolic reactions is affected by stress conditions including acid, high osmotic pressure, heavy metals, and strong oxidants such as ferric ion, and can be measured to determine which metabolites are undergoing major changes, providing information that are relevant to the understanding of the physiological changes related to the industrial process and the environment. This type of analysis, combining adverse conditions such as toxicity and microbial metabolic activity, has been performed in different organisms.

While there are very few studies in biomining microorganisms, there are some interesting examples in other bacterial genera such as *Pseudomonas*, *Streptococcus*, and *Actinomyces*, among others, where the use of metabolomics tools has provided a comprehensive understanding of the biofilm formation and its implications (Gjersing et al. 2007; Takahashi et al. 2012; Zhang and Powers 2012). Recently, Mosier et al. (2013) conducted an interesting study of metabolites associated with adaptation of microorganisms to acidic and metal-enriched conditions. A remarkable feature in this study is the analysis of microorganisms under natural conditions, where it was feasible to study members of an ecosystem that may not necessarily be cultured in the laboratory, an approach analogous to metagenomics studies. Although the complexity of acidic ecosystems and technical difficulties for metabolite extraction from the environmental matrix complicate the data analysis, the authors used tools which facilitate data collection, such as application of stable isotopes, untargeted analysis, and measurement by mass spectrometry (MS), all in order to confirm the physiological significance of detected metabolites. Samples of natural biofilms were obtained from AMD (Richmond Mine, Iron Mountain, California), categorized according to stage of development, and cultured in the laboratory in the presence of ^15^N-labeled ammonium sulfate in order to identify metabolites and the number of nitrogen atoms present. The composition of the biofilm was determined by fluorescence in situ hybridization, which identified mostly members of *Leptospirillum* (groups II and III), Archaea, and (in lesser amounts) *Sulfobacillus*. Metabolites were extracted and analyzed by liquid chromatography coupled to MS, which detected 3500 ions with retention times and mass/charge values. The use of blank and control conditions without ^15^N eliminated the inherent noise of these extremely sensitive technologies. Among these ions, 241 were released, confirming that 80 % respond to criteria of specific technical modifications (e.g., adduct ion fragmentation). After manual depuration, 56 metabolites were identified and associated with chemical formulas. However, the results indicated that more than 90 % of the metabolites were unknown and did not match any MS/MS spectra found in the databases (clearly biological “dark matter”). It is important to remark that metabolite databases are associated with commercial standards that are estimated to represent only half of the existing biological metabolites (García et al. 2008). Of the metabolites identified, three are likely to be significant in biofilms: phosphatidylethanolamine lipids, taurine, and hydroxyectoine. The phosphatidylethanolamine lipids detected had been reported in previous studies of acidophilic microbial communities (Fischer et al. 2012) and were associated with *Leptospirillum* group II based on correlations of lipid and protein abundance. An interesting role has been suggested by this type of molecule in the biofilm: to be involved in a potential mechanism of resistance to toxic elements present in AMD solutions with a similar composition that the ones found under industrial bioleaching conditions (Druschel et al. 2004). Phosphatidylethanolamine has also been found in eukaryotes, such as the acidophilic fungus *Acidomyces richmondensis*, an organism found in abundance in AMD. Genetic studies of this microorganism demonstrated the presence of eight genes involved in synthesis of this molecule, suggesting its production in biofilms associated with bacteria and fungi. An additional metabolite found was taurine (Mosier et al. 2013). This molecule has been widely studied and reported to have multiple functions in different microorganisms (Huxtable 1992). It is utilized as the sole source of carbon, nitrogen, and sulfur and as an osmotic regulator, when microorganisms are exposed to high ion concentrations typical of biomining processes. With this information, the authors analyzed the genome sequences of microorganisms present in these environments in the search of genes related to taurine synthesis. It was found that neither Bacteria nor Archaea could synthesize taurine, but that sulfobacilli possesses transporters which could allow taurine uptake into the cell in response to unfavorable environmental conditions. Moreover, *A. richmondensis* could be responsible for taurine synthesis and degradation in biofilms, as the genes involved in taurine synthesis were found to be encoded in its genome. It is noteworthy that the authors were unable to identify taurine in pure cultures of this organism by MS, possibly owing to unfavorable conditions for taurine synthesis in laboratory or a potential cooperative role typical in this kind of complex ecosystem. A third metabolite detected in this study is the hydroxyectoine, which was detected in natural biofilms as well as in laboratory-grown enriched biofilms. This molecule may act as a compatible solute in the adaptation to hyperosmotic stress, which can provide resistance to high temperatures, dehydration, and freezing. The genome of *Leptospirillum* group II has the genetic components for synthesis of this metabolite and a similar metabolite, ectoine, that was also detected (not confirmed by MS/MS). This observation was corroborated by a previous proteomic analysis (Mueller et al. 2011) that detected proteins related to hydroxyectoine synthesis, especially in the early stages of biofilm formation. Other members of the biofilm community also have genes encoding for ectoine synthesis, including *Ferroplasma* and *Sulfobacillus* genera. These observations slowly start to contribute to our understanding of the metabolic dynamics of biomining acidophiles exposed to extreme conditions. Some osmoprotectant metabolites are synthesized in greater quantities in the early stages of colonization of a mineral compared with late stages, supporting the hypothesis that microorganisms are more exposed to high ion concentrations in their planktonic state than in biofilms. Several of these of metabolites, generated in response to adverse conditions in an ecosystem, are good candidates to be used as biomarkers in industrial processes as indicators of cell activity in biomining applications.

Our research group has recently published the first metabolomic study in Bacteria using two microorganisms isolated from mining sites in Chile, *Acidithiobacillus ferrooxidans* strain Wenelen, DSM 16786 and *Acidithiobacillus thiooxidans* strain Licanantay, DSM 16786 (Martínez et al. 2013). Both have been extensively studied and are recognized as having a key role in the bioleaching process. They have been shown to possess improved oxidizing activities compared with other known microbes from the same species, which has allowed their use in biomining processes that optimize copper recovery from copper sulfide ores (Sugio et al. 2009; Ohata et al. 2010). This study employed both targeted and untargeted analytical strategies for analysis and detection of metabolites, using capillary electrophoresis and MS for separation and identification, respectively. The metabolomic analysis was performed after exposing microorganisms to different energy sources, such as iron, sulfur, and copper sulfide mineral chalcopyrite (CuFeS_2_). The two latter substrates correspond to insoluble energy sources, which allowed analysis of metabolism in planktonic as well as biofilm cells. Samples were collected at different stages of growth, and intracellular and secreted metabolites were analyzed. The overall objective of this work was to extend the functional knowledge of these microorganisms based on the search for active pathways that may be key to cell physiological processes under different growth conditions. There were clear differences between metabolic profiles of microorganisms exposed to different energy growth conditions. In addition, differences between metabolic profiles of attached and free microorganisms were detected. Metabolomic analysis using standards for metabolite identification (targeted metabolomics) provided interesting results on metabolic pathways that may play a major role in bioleaching. Synthetic pathways for polyamine, GSH, and amino acids and metabolites involved in energy processes were highlighted and analyzed in greater detail. The results indicate that GSH synthesis routes and some of its precursors, such as glutamate, were overexpressed in both species when grown in the presence of sulfur, a phenomenon not observed when the microorganisms were grown in the presence of iron or chalcopyrite. This observation is consistent with the previously described role of GSH in sulfur oxidation in this type of microorganisms (Rohwerder and Sand 2003). Significant differences were also found in amino acid synthesis under various growth conditions for each microorganism. In *Acidithiobacillus thiooxidans* strain Licanantay, the amino acid differences observed between sulfur and chalcopyrite conditions could be explained by differential synthesis of proteins required for adaptation to a new energy source and specific detoxification mechanisms under each condition. A similar phenomenon was found in *Acidithiobacillus ferrooxidans* strain Wenelen grown with ferrous iron versus chalcopyrite. While this comparative analysis seems to be useful, its future integration with proteOMIC data is a must. An interesting observation was the abundant detection of certain amino acids (such as aspartate and glutamate) in the extracellular space. This phenomenon has been reported in other organisms (e.g., *Bacillus subtilis*) which secrete these amino acids to produce polyglutamate and polyaspartate related to biofilm synthesis (Morikawa et al. 2006); however, such polymeric structures were not detected surrounding these biomining microorganisms. Another possibility is that secretion of these amino acids is related to an intracellular accumulation phenomenon or disturbances in cytoplasmic membranes, as reported before (Hoischen and Krämer 1990). Further investigation is needed to determine the physiological significance of this observation and any potential applications. An additional interesting observation in this study was the abundant detection of the intracellular carbohydrates sedopheptulose-7-phosphate and dihydroxyacetone phosphate, both in *Acidithiobacillus thiooxidans* strain Licanantay when grown in sulfur. These compounds are linked to formation of large structures such as EPS involved in biofilm synthesis, and may be correlated with previously described genomics studies. Metabolites related to energy metabolism pathways (e.g., synthesis of NADP, AMP, ADP) were produced in greater abundance by *Acidithiobacillus thiooxidans* strain Licanantay than *Acidithiobacillus ferrooxidans* strain Wenelen when both were grown in sulfur. This is consistent with the improved efficiency of *Acidithiobacillus thiooxidans* for processing of sulfur (Knickerbocker et al. 2000). Another pathway highlighted in this study is polyamine synthesis. Polyamines are polycationic compounds present in all cells, specifically found in the intracellular space (Tabor and Tabor 1985), and involved in a variety of biological responses such as cellular proliferation, differentiation, biofilm formation (Igarashi and Kashiwagi 1999), protein synthesis (Friedman and Oshima 1989), and DNA synthesis and stabilization (Terui et al. 2005). Different kinds of polyamines have been reported, the most common being putrescine, spermidine, and spermine. The study analyzed the polyamine pathway in both strains, and found that levels of S-adenosyl-l-methionine, a spermidine synthesis intermediate, were below the detection limit in early growth stages but elevated in exponential and stationary phases under all growth conditions (sulfur, iron, and chalcopyrite). Nevertheless, spermidine, which was also present in bacterial supernatants, was detected at levels 5- to 6-fold higher with respect to *S*-adenosyl-l-methionine during growth phases, indicating a tendency for its accumulation during growth. Interestingly, intracellular levels of spermidine in *Acidithiobacillus ferrooxidans* strain Wenelen were approximately one third higher in iron-containing medium compared with sulfur-containing medium. In contrast, data from cells grown in sulfur-containing medium with solid surface stimuli show reduced intracellular accumulation and the presence of extracellular spermidine. For *Acidithiobacillus thiooxidans* strain Licanantay, it was expected that both solid energy sources (sulfur and chalcopyrite) would enhance biofilm production and reveal similar tendencies with respect to spermidine secretion and concentration. Surprisingly, spermidine secretion was observed only in sulfur-grown cells. The reason for this phenomenon is not clear but may be related to an efficient use of this energy source. Two additional intermediates in the spermidine biosynthesis pathway, arginine and ornithine, showed opposite behaviors: arginine production increased, whereas ornithine decreased over time. Common polyamine synthesis routes such as putrescine, spermidine, and spermine have been described in bacteria, with ornithine and arginine used as precursors. Additionally, a variety of new polyamines and biosynthesis routes have been described, mainly in thermophiles from the Archaea and Bacteria domains (Ohnuma et al. 2005; Terui et al. 2005). The metabolomic findings extracted from this work suggest the possible existence of an alternative route for polyamine synthesis, different from the canonical pathway. The common bacterial spermidine intermediate, putrescine, was not detected in any condition, suggesting that spermidine synthesis might be analogous to routes described in other extremophiles (Ohnuma et al. 2005). However, a MS-based search of some of the intermediates reported in these alternative routes was not successful in either organism, suggesting the presence of alternative molecules for this metabolic pathway in *Acidithiobacillus thiooxidans*. The observation that spermidine is secreted only under sulfur-growth conditions suggests that it could be acting as a communication molecule for bacterial adhesion and biofilm formation on hydrophobic energy substrates such as sulfur, as suggested before (Karatan et al. 2005; Sturgill and Rather 2004). As mentioned previously, biofilm formation in *Acidithiobacillus ferrooxidans* is closely related to production of extracellular components such as EPS, and biofilm composition changes according to the energy substrate and differential gene expression (Barreto et al. 2005; Gehrke et al. 1998). This appears to be consistent with the abundant spermidine found in *Acidithiobacillus ferrooxidans* strain Wenelen and *Acidithiobacillus thiooxidans* strain Licanantay supernatants, particularly in sulfur-growth conditions. A significant impact of these metabolOMIC finding has allowed the proposal that spermidine can be used as a biomarker for sulfur-oxidizing activity in bioleaching processes (Martínez and Parada 2012).

Additionally, currently in our research group metabolomic studies on bacterial isolates of the genus *Leptospirillum* and Archaeal isolates from the genus *Ferroplasma* have been conducted. Both iron-oxidizing microorganisms have shown the capability to secrete molecules from the flavin family (enzyme cofactors) to the external medium (Martínez, unpublished data). This phenomenon could be related to the mechanism of electron transfer from Fe(II) into the cell, similar to that reported in the iron reducer *Shewanella oneidensis* (Kotloski and Gralnick 2013). These molecules are distinct in each organism, so that they can also be used as candidates for specific biomarkers.

All of these studies provide examples of how the knowledge extracted with metabolomics can be used to understand the role of key metabolic pathways in acidophiles, with an enormous potential to be applied in industrial operations.

## Conclusions

Through this mini-review, the current state of genomics, proteomics, and metabolomics of bioleaching has been briefly revised, attempting to show the major impacts of these analytical methods at industrial-scale bioleaching. Genomics has currently impacted the determination of leaching biodiversity and the development of metabolic models. Metagenomics is called to impact the field in the coming future by means of the analysis of spatial and temporal microbial population dynamics during bioelaching stages using next-generation sequencing technologies, as well as metabolic functional models, allowing optimal bioleaching operational design and control.

Proteomics future impacts demand major efforts to unravel the functional characterization of the identified proteins, including the hypothetical and with unknown function ones, which in some cases may account for more than 20 % of the total detected proteins in high-throughput studies. Future attempts to understand biofilm formation and development of relevant industrial strains, and their interaction with mineral surfaces in mixed species cultures, as well as development of biomarkers to analyze the microbial biodiversity within field operations are major challenges which will enhance our knowledge for future bioleaching processes development and monitoring.

Last but not least, metabolomics is clearly on the first stages of development in the field of bioleaching. However, the discovery of unique metabolites of acidophiles with biotechnological potential, as well as the use of specific metabolites as biomarkers to assess the microbial activity within bioleaching processes, highlights its future industrial application.
